# Comparing performances of different statistical models and multiple threshold methods in a nested association mapping population of wheat

**DOI:** 10.3389/fpls.2024.1460353

**Published:** 2024-10-01

**Authors:** Karansher S. Sandhu, Adrienne B. Burke, Lance F. Merrick, Michael O. Pumphrey, Arron H. Carter

**Affiliations:** Department of Crop and Soil Sciences, Washington State University, Pullman, WA, United States

**Keywords:** false positives and false negatives, genome-wide association studies, nested association mapping, single and multi-locus models, spectral reflectance indices, wheat

## Abstract

Nested association mapping (NAM) populations emerged as a multi-parental strategy that combines the high statistical power of biparental linkage mapping with greater allelic richness of association mapping. Several statistical models have been developed for marker-trait associations (MTAs) in genome-wide association studies (GWAS), which ranges from simple to increasingly complex models. These statistical models vary in their performance for detecting real association with the avoidance of false positives and false negatives. Furthermore, significant threshold methods play an equally important role for controlling spurious associations. In this study, we compared the performance of seven different statistical models ranging from single to multi-locus models on eight different simulated traits with varied genetic architecture for a NAM population of spring wheat (*Triticum aestivum* L.). The best identified model was further used to identify MTAs for 11 different agronomic and spectral reflectance traits, which were collected on the NAM population between 2014 and 2016. The “Bayesian information and linkage disequilibrium iteratively nested keyway (BLINK)” model performed better than all other models observed based on QQ plots and detection of real association in a simulated data set. The results from model comparison suggest that BLINK controls both false positives and false negatives under the different genetic architecture of simulated traits. Comparison of multiple significant threshold methods suggests that Bonferroni correction performed superior for controlling false positives and false negatives and complements the performance of GWAS models. BLINK identified 45 MTAs using Bonferroni correction of 0.05 for 11 different phenotypic traits in the NAM population. This study helps identify the best statistical model and significant threshold method for performing association analysis in subsequent NAM population studies.

## Introduction

Connecting phenotypes with genotypes provides a vital tool for crop breeding and improvement. The most commonly exploited approaches for genetic mapping include biparental linkage mapping and association mapping. Biparental linkage mapping requires developing a large recombinant population for linkage-based mapping of quantitative trait loci (QTLs) (Lander and Botstein, 1989). Methods for linkage mapping range from single marker analysis to more sophisticated methods such as interval mapping, composite interval mapping, multiple regression, and joint mapping (Li et al., 2010). However, the excitement of linkage mapping was surpassed by association mapping in the mid-2000s because of its two main advantages. First, association mapping does not require the time, cost, and effort needed to create recombinant inbred lines. Second, association mapping provides a high resolution compared to biparental mapping, as they have numerous crossover events (Zhu et al., 2008). However, association mapping has low power for rare alleles compared to linkage mapping; that is why both techniques are not separated from each other and are used interchangeably (Bernardo, 2008).

Nested association mapping (NAM) populations emerged as a multi-parental strategy that combines the high statistical power of biparental linkage mapping with greater allelic richness of association mapping (Yu et al., 2008). NAM provides a higher genetic variation, reduces linkage disequilibrium, increases mapping resolution, and reduces population structure (Yu et al., 2008). Even though population structure is typically ignored due to genome reshuffling, it can still be accounted for in the model for controlling spurious association (McMullen et al., 2009a). Analysis of NAM populations has yielded high confidence for marker-trait association (MTAs) in maize (*Zea mays* L.) (McMullen et al., 2009b), soybean (*Glycine max* L.) (Song et al., 2017), and barley *(Hordeum vulgare* L.) (Nice et al., 2017). MTAs for a NAM population can be performed with joint linkage association mapping and genome-wide association studies (GWAS) (Kaur et al., 2021). Joint linkage association mapping involves nesting the QTL term within families allowing the differentiating of allelic variations from linked genes (Li et al., 2011).

Several statistical models have been developed for MTAs in GWAS, which range from simple to increasingly complex (Liu et al., 2016). With an increase in genotyping information, statistical models that can separate the real biological association from false positives are required without comprising real association (false negatives). False positives in models are also observed when familial relatedness or common ancestry between the genotypes is not accounted for. Structure, discriminant analysis, and principal components analysis (PCA) are routinely used as a covariate in statistical models to account for population structure (Pritchard et al., 2000; Price et al., 2006). However, PCA is getting more attention because of its consistent performance with structure, and it is computationally cheap to generate the covariates (Wang et al., 2009; Wu et al., 2011). Identity by descent is one of the traditionally used approaches for observing familial relatedness. Recently, the kinship matrix calculated from genotyping information has been used as a covariate in the mixed linear models (MLMs) (VanRaden, 2008).

Most simple GWAS analysis involves a general linear model (GLM) where single nucleotide polymorphism (SNP) and population structure are used as a fixed effect, but GLM results in a large number of false positives (Singh et al., 2023). The MLM was further developed to include population structure as a fixed effect and kinship as a random effect in the model to control for false-positive associations (Yu et al., 2006). The MLM treats each individual separately in the model, making it computationally very expensive. The compressed mixed linear model (CMLM) was developed to decrease the computational time of MLM by grouping individuals in the random effect model and has similar or higher statistical power than MLM (Zhang et al., 2010). These models use all the set of SNPs in the analysis, but the “settlement of MLM under progressively exclusive relationship (SUPER)” model extracts a small number of SNP called pseudo quantitative trait nucleotide (QTN) for obtaining kinship, giving higher power and being less computationally intensive than previous models (Wang et al., 2014).

One thing about all these GWAS models is that they are single-locus models, which include scanning one marker at a time, and this process is repeated iteratively for each marker. These single-locus models fail to imitate the real genetic makeup of complex traits, which are controlled by a large number of QTLs and their interactions. Segura et al. (2012) solved this issue with the inclusion of multi-locus association analysis using heritable variance estimate criteria for inclusion of factors in the forward and backward elimination in the multi-locus mixed model (MLMM). This model is shown to induce false negatives because of overfitting in the model and missing the true associations (Liu et al., 2016). A “fixed and random circulating probability unification model (FarmCPU)” is a novel multi-locus model controlling both false negatives and false positives while being computationally very efficient.

Recently, the “Bayesian information and linkage disequilibrium iteratively nested keyway (BLINK)” model was developed, which combines better computational efficiency with higher statistical power (Huang et al., 2018). This model avoids the biggest limitation of all the mixed models which assumes that causal genes are distributed uniformly over the genome, which usually results in the inclusion of non-causal genes or the missing of two causal genes present in the same bin (Huang et al., 2018). Contrastingly, BLINK works directly based on linkage disequilibrium (LD) information compared to bin method. Furthermore, this model uses Bayesian information content to obtain the maximum likelihood for selecting the associated markers. As both the testing of markers and selecting associated markers are performed as a cofactor in the fixed effect model, there is a significantly higher computational advantage provided with this model (Huang et al., 2018).

False negatives cannot only arise because of overfitting in the model but can also be due to over conservative thresholds applied for determining the significant threshold (Pe’er et al., 2008; Dudbridge and Gusnanto, 2008). The most commonly used multi-comparison thresholds in GWAS studies are the false discovery rate (FDR), positive FDR, and Bonferroni correction (Holm, 1978; Benjamini and Hochberg, 1995; Johnson et al., 2010). Therefore, selecting the appropriate threshold and model for association studies is essential for controlling both false negatives and false positives.

The objectives of this study were to compare seven different GWAS models varying from single to multi-locus in a spring wheat NAM population. The model’s performances were validated on eight different simulated traits having varied genetic architecture. The NAM population was selected in this study to identify the most appropriate GWAS model. We also evaluated four different multi-comparison methods for identifying significant associations. Finally, we identified the MTAs for 11 different phenotypic traits collected from the NAM population using the best identified model.

## Materials and methods

### Plant material

The dataset used in this study consisted of a three-year (2014–2016) field trial of 650 recombinant inbred lines (RIL) from a NAM population described previously (Sandhu et al., 2021d). The complete information about parents, population development, and field trial is referred to in previous publications (Sandhu et al., 2021a, Sandhu et al., 2021c; Blake et al., 2019; Jordan et al., 2018). Briefly, the NAM population consisted of 26 founder parents crossed to a common cultivar Berkut to obtain the population. A modified augmented design was used for planting 650 RILs at Spillman Agronomy Farm near Pullman, WA. Five different data points, namely, plant height (PH), days to heading (DTH), test weight (TSTWT), grain protein content (GPC), and grain yield (GY) were collected. In addition, six spectral reflectance indices (SRIs) were obtained using a handheld CROPSCAN multi-spectral radiometer (CROPSCAN, Inc. Rochester, MN, USA) at the grain-filling stage. These indices were normalized difference vegetation index (NDVI), normalized water index (NWI), photochemical reflectance index (PRI), anthocyanin reflectance index (ARI), normalized chlorophyll pigment ratio index (NCPI), and green normalized difference vegetation index (GNDVI). The complete details about the collection of spectral information and extraction of these indices can be referred to in Sandhu et al. (2021a, c).

### Statistical data analysis

Best linear unbiased predictors (BLUPs) for all the traits were obtained using an augmented complete block design (ACBD) model implemented in R (Rodríguez et al., 2018; R Core Team, 2020). BLUPs were obtained for combined analysis across the environments using the model:


$${\text{Y}}_{\text{ijkl}}=µ+\text{ Bloc}{\text{k}}_{\text{i}}+Gen_{j}+\text{Chec}{\text{k}}_{\text{k}}+\text{En}{\text{v}}_{\text{l}}+Env_{l}⊗Gen_{j}+e_{ijkl}$$

where Y_ijkl_ is the trait of interest, µ is the mean effect, Block_i_ is the fixed effect of the ith block, Gen_j_ is the random effect of unreplicated genotypes *j* and distributed as independent and identically distributed, Gen_j_ ~ N(0, σ^2^_g_), Check_k_ represents the fixed effect of each replicated check cultivar in the block, Env_l_ is the fixed effect of the lth environment, Env_l_ ⨂ Gen_j_ are the random effects of environment and genotype interaction, and e_ijkl_ is the standard normal error distributed as e_ij_ ~ N(0, σ^2^_e_).

Broad sense heritability of each trait was calculated across the environments using the formula:


$$H^{2}=1-\;{{\bar{v}}_{\text{Δ}.}}^{BLUP}/2{\text{σ}}^{2}\text{g}$$

where H^2^ is broad sense heritability, σ^2^_g_ is the genotypic variance, and ${{\bar{v}}_{\text{Δ}.}}^{BLUP}$ is mean variance of the BLUPs (Cullis et al., 2006).

### Genotypic data and population structure analysis

The whole NAM population was genotyped with the Illumina 90K SNP array and genotyping by sequencing (Wang S. et al., 2014; Poland et al., 2012). The complete set of markers consisted of 73,345 polymorphic markers anchored to the Chinese Spring RefSeqv1 (Jordan et al., 2018). Detailed procedures about genotyping, marker calling, and map construction are reported previously Sandhu et al., 2021b; Sandhu et al., 2021). Quality control was performed where RILs missing phenotyping data were removed from the analysis. Markers with minor frequency less than 0.10 had missing data more than 20%, or were monomorphic, and RILs missing more than 10% of the genotypic data were discarded for further analysis. In the end, we were left with 635 RILs with 44,000 SNP markers. The PCA was performed for assessing the population structure present in the population using the whole set of 44,000 SNP markers using “prcomp” function in R (R Development Core Team, 2020; Price et al., 2006).

### Simulation of traits

We simulated eight different traits with varying heritability and number of QTLs. These traits were simulated by using the genotypic information described above in R using “GAPIT” and “bigmemory” packages (Lipka et al., 2012; R Development Core Team, 2020). The traits were simulated to mimic the trait having a particular heritability and genetic architecture. These simulated traits were generated using the same genotypic markers that were used for NAM population. These data were simulated to have random QTL effects. The simulated traits were having H^2^ = 20% and QTLs = 10 (H20_Q10), H^2^ = 40% and QTLs = 10 (H40_Q10), H^2^ = 60% and QTLs = 10 (H60_Q10), H^2^ = 80% and QTLs = 10 (H80_Q10), H^2^ = 20% and QTLs = 20 (H20_Q20), H^2^ = 40% and QTLs = 20 (H40_Q20), H^2^ = 60% and QTLs = 20 (H60_Q20), and H^2^ = 80% and QTLs = 20 (H80_Q20). The whole NAM population was simulated for having a random QTL effect with different heritabilities.

### GWAS models

We evaluated seven different GWAS models for association analysis in a NAM population. These models varied from simple to increasingly complex, namely, (a) GLM (Price et al., 2006), (b) MLM (Yu et al., 2006), (c) CMLM (Zhang et al., 2010), (d) SUPER (Wang et al., 2014), (e) MLMM (Segura et al., 2012), (f) FarmCPU (Liu et al., 2016), and (g) BLINK (Huang et al., 2018).

GLM is the simplest GWAS model fitting only population structure and testing each SNP one at a time in the fixed effect model. The GLM can be represented as


Y=SNP+Q[PCs]+e

where Y is a matrix of phenotypic information, SNP represents the matrix of markers, Q represents the population structure, and e is the residual error (Price et al., 2006).

GLM results in false positives, and this was avoided by MLM with inclusion of family relatedness in the model. MLM includes the kinship matrix as random effect in the mixed effect model and MLM can be represented as


Y=SNP+Q[PCs]+Kinship+e

Kinship represents the relationship matrix between the individuals included in the model. All other variables of this equation are described above. SNP and Q are set as fixed effects, while kinship is random effect in the model (Yu et al., 2006).

MLM was computationally very intensive, because computational time varies with the third power of number of individuals in the random effect model. Furthermore, there were confounding issues between testing marker, structure, and kinship matrix as same set of markers were double counted. CMLM clustered the individuals into different groups resulting in a reduction of effective size of random effect model (Zhang et al., 2010). CMLM obtains the kinship among the groups and is computationally more efficient than MLM. CMLM can be represented as


Y=SNP+Q[PCs]+Kinship+e

where kinship is the relationship matrix among the groups and other terms are the same as MLM described above.

The SUPER model uses only associated markers known as QTN for calculating the complementary kinship with exclusion of the associated QTN. This model removes the individual markers from the kinship if the testing marker is in LD with it regardless of the physical distance between them (Wang et al., 2014). This model is shown to provide higher computational power using a set of QTN for obtaining the kinship and can be represented as


Y=SNP+Q[PCs]+Complementary kinship+e

where complementary kinship is obtained using pseudo QTN, and other terms are same as MLM described above.

All the above models are single locus models, which are not appropriate for most of complex traits in the wheat. MLMM includes testing of multiple markers at a time, and it is more important for wheat as confounding effects are present across the genome. MLMM uses forward inclusion of markers using heritable variance as an estimate to stop the inclusion of the marker. Then backward elimination conducted from the last forward model is used to completely explore the model space. The MLMM can be represented as


Y=SNP+QTN1+QTN2+QTNn+ Q[PCs]+Kinship+e

where QTN1 to QTNn represents the pseudo QTN included in the model using forward inclusion and backward elimination. All other terms of the equation are similar to MLM.

MLMM tests multiple markers as covariates in stepwise regression to remove the confounding effects between markers and kinship. To fully remove the confounding problem, MLMM is divided into fixed and random effect models in the FarmCPU model. The fixed effect model tests single markers at a time with multiple associated markers as a covariate to control for false positives. Furthermore, model overfitting is avoided in the random effect model by obtaining kinship using multiple associated markers. The *p*-value of each tested and associated marker is unified at each iteration. The FarmCPU model can be represented as


Y=SNP+QTN1+QTN2+QTNn+ Q[PCs]+e

This is the fixed effect component of the FarmCPU model, with individual markers tested one at a time and other terms of the equation as described previously.


Y=Q[PCs]+Kinship+e

This is the random effect component of the FarmCPU model, and all terms of the equation are described previously.

FarmCPU has an efficient fixed model, but it has computationally expensive random effect model. Furthermore, QTNs in random effect models were selected based on their even distribution over the genome. To increase the computational efficiency, the random effect model was replaced with a fixed effect model using Bayesian information criteria. This new method is known as “BLINK” as QTNs were selected based on the linkage disequilibrium information.

### Interpretation of Q-Q plots and simulated data output

False positives and false negatives can be interpreted by looking at the Q-Q plots generated by the model. The Q-Q plots depict the observed negative log of association of probability across all markers (*y*-axis) to the expected negative log of association probability (*x*-axis). If the Q-Q plot has a straight line with 1:1 with absence of any tail, this suggests that data follow the normal distribution and the null hypothesis is true, meaning there is no true or significant association (Würschum et al., 2012; Stich et al., 2008). If the line does not follow 1:1, it suggests that null hypothesis is not true and there are significant associations present. In the case the Q-Q plot has an upward inflation, there are false positives, while downward inflation depicts presence of false negatives. On the other hand, if the Q-Q plot has a straight 1:1 line with a sharp upward tail at the end, this suggests that both false positives and false negatives are controlled, and only real associations are visible. This happens because most of the *p*-values follow a uniform distribution, while few have deviations, suggesting that those associations are in linkage disequilibrium with the causal polymorphism (Würschum et al., 2012; Stich et al., 2008). Furthermore, output of each model was used to compute the true associations, false positives, and false negatives associations under each simulation scenario to assess the performance.

### Models evaluation

Model performances were evaluated by looking at the Q-Q plots for identifying the false positives and false negatives. Models were also evaluated by looking at the simulated data to observe how many simulated QTNs are detected by the models. Furthermore, false positives and false negatives were identified using each model for assessing their performances. The best identified model was later used for MTAs for 11 phenotyping traits collected from the NAM population.

### Evaluation of multiple comparisons methods for significant threshold

We evaluated four different multiple comparisons methods for determining the significant threshold to determine association. These methods were the FDR, Bonferroni correction, positive FDR, and log_10_3. All these comparisons were made using multiple methods comparison methods in JMP Genomics 6.0 (SAS Institute Inc, 2011).

The FDR method, based on the Benjamini–Hochberg procedure, controls the expected proportion of false positives among the declared significant results. It calculates the significance threshold by adjusting the *p*-values according to their rank (k) relative to the total number of tests (m) and the desired significance level (α), using the equation:


FDR=k/m×α

The Bonferroni correction is a more conservative approach that controls the family-wise error rate by dividing the significance level by the number of comparisons (m), resulting in an adjusted threshold (α):


α′=α/m

The positive FDR (pFDR) method focuses on the proportion of false discoveries among all discoveries, conditional on there being at least one discovery. The pFDR is calculated using the expectation of false positives (V) relative to the number of rejected hypotheses (R):


pFDR=E[V∣R>0]/R

Finally, the log10-transformed *p*-values method provides an alternative way to interpret small *p*-values by transforming them using the log base 10. The transformed *p*-value (plog10p_{\text{log10}}plog10​) is calculated as:


plog10=−log10(p)

This transformation allows for easier interpretation of extremely small *p*-values, though it does not directly control for multiple comparisons.

## Results

### Phenotypic data summary

The 11 different traits used in this study have a broad phenotypic range as observed (**Table 1**), which is relevant for performing association analysis. Broad sense heritability ranged from 34% to 90% providing detailed variation for dissecting the model’s performance. All these traits have different genetic architecture, which will help validate the performance of GWAS models using Q-Q plots to identify false positives and false negatives.

**Table 1:** Phenotypic information and broad sense heritability for 11 different traits collected from a nested association mapping population of spring wheat planted in the U.S. Pacific Northwest.

	GY (t/ha)	GPC (%)	TSTWT (kg/hl)	PH (cm)	DTH	NDVI	NWI	GNDVI	PRI	NCPI	ARI
Mean	2.08	13.03	73.97	92.28	169.1	0.72	0.06	0.68	−0.12	0.34	1.13
Minimum	1.00	11.22	69.13	77.70	163.9	0.51	0.03	0.55	−0.18	0.19	0.11
Maximum	2.82	15.61	77.62	109.80	178.1	0.86	0.09	0.77	−0.09	0.48	1.71
Heritability	0.34	0.66	0.69	0.72	0.90	0.74	0.67	0.71	0.69	0.55	0.61

GY is grain yield, GPC is grain protein content, TSTWT is test weight, PH is plant height, DTH is days to heading, NDVI is normalized difference vegetation index, NWI is normalized water index, GNDVI is green normalized difference vegetation index, PRI is photochemical reflectance index, NCPI is normalized chlorophyll pigment ratio index, and ARI is anthocyanin reflectance index.

### Model comparisons with simulated data set using Q-Q plots and true associations

**Figure 1:**
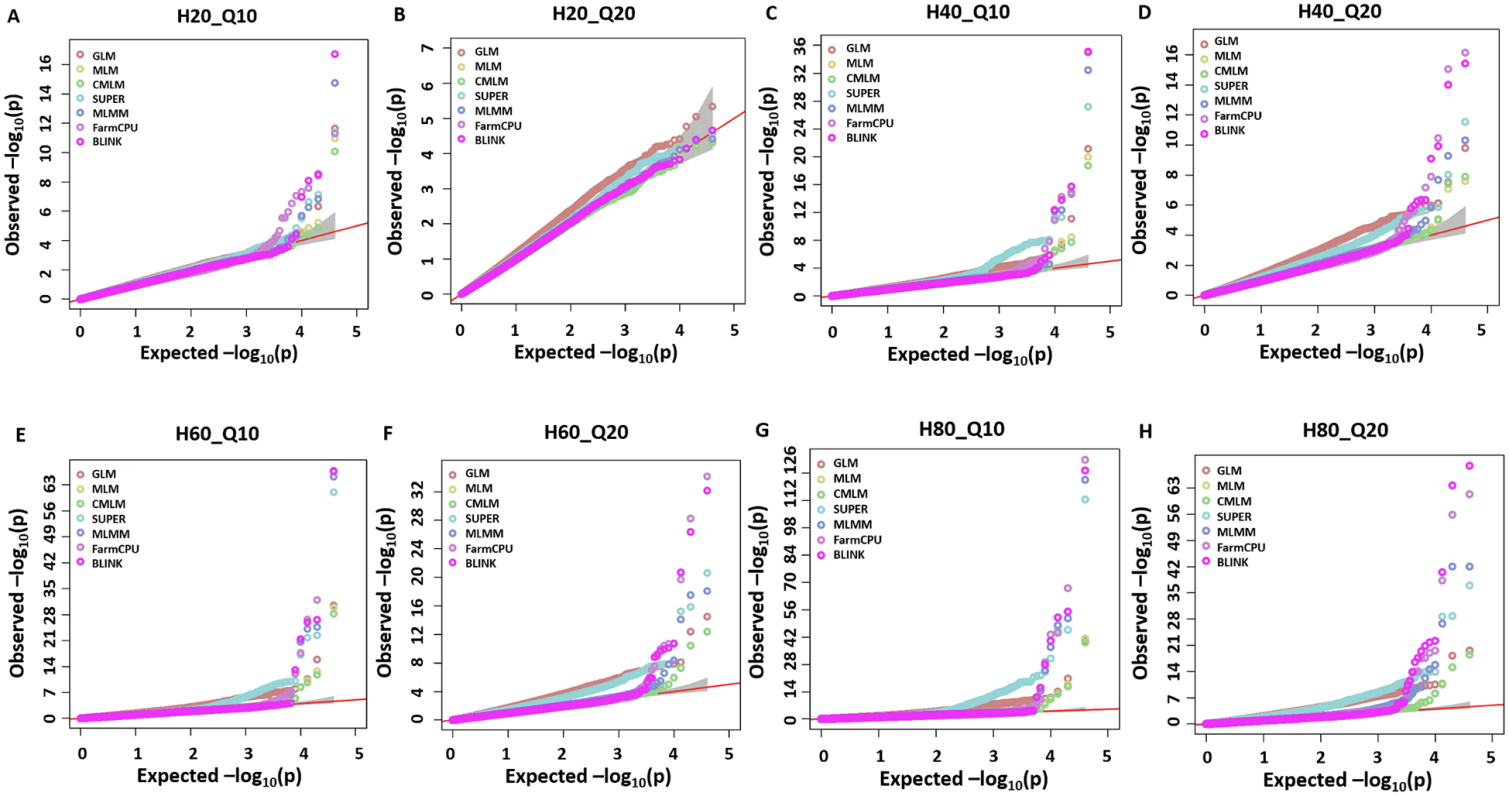
Q-Q plots for the seven different GWAS models, namely, general GLM, MLM, CMLM, SUPER, MLMM, FarmCPU, and BLINK for comparing performances of eight simulated traits including H = 20% and Q = 10 (H20_Q10) **(A)**, H = 20% and Q = 20 (H20_Q20) **(B)**, H = 40% and Q = 10 (H40_Q10) **(C)**, H = 40% and Q = 20 (H40_Q20) **(D)**, H = 60% and Q = 10 (H60_Q10) **(E)**, H = 60% and Q = 20 (H60_Q20) **(F)**, H = 80% and Q = 10 (H80_Q10) **(G)**, and H = 80% and Q = 20 (H80_Q20) **(H)** for a nested association mapping population of wheat.

Seven different GWAS models which varied from simple to complex were used to compare their performances over the simulated dataset of a NAM population. The Q-Q plots for eight different simulated traits with seven GWAS models are provided in **Figure 1**. Among the seven evaluated GWAS models, BLINK performed best regarding control of false positives and false negatives. In the case of H20_Q10, all models performed similarly because of the small amount of genetic variation (**Figure 1A**) (**Table 2**). We showed the results for the true associations, false positives, and false negatives associations by each model for H20_Q10 scenario (**Table 2**). As we move towards H80_Q20, a clear difference in performance is seen for each model (**Figure 1**) (**Table 2**). GLM, SUPER, and FarmCPU result in false positives for all eight simulated traits as evident from slightly deviated tails compared to the straight 1:1 line for BLINK (**Figure 1**). Furthermore, when the number of simulated QTLs increased to 20, MLM, CMLM, and MLMM also showed an inflation of false positives in addition to GLM, SUPER, and FarmCPU (**Figures 1B, D, F, H**). We showed the results for the true associations, false positives, and false negatives associations by each model for H80_Q20 scenario (**Table 2**). This further strengthens that the recently developed model BLINK performed superior in controlling false positives for the tested spring wheat NAM population.

**Table 2:** True associations, false positives and false negatives association detected using seven different GWAS models namely general GLM, MLM, CMLM, SUPER, MLMM, FarmCPU, and BLINK for H20_Q10 and H80_Q20 simulated scenario.

		Model type						
		GLM	MLM	CMLM	SUPER	MLMM	FarmCPU	BLINK
H20_Q10	True associations	2	1	1	1	2	3	4
	False positives	2	0	0	3	0	1	0
	False negatives	8	9	9	9	8	7	6
H80_Q20	True associations	8	5	5	13	11	13	15
	False positives	3151	0	0	869	0	2	0
	False negatives	12	15	15	7	9	7	5

### Model comparisons with simulated data set using Manhattan plots

**Figure 2:**
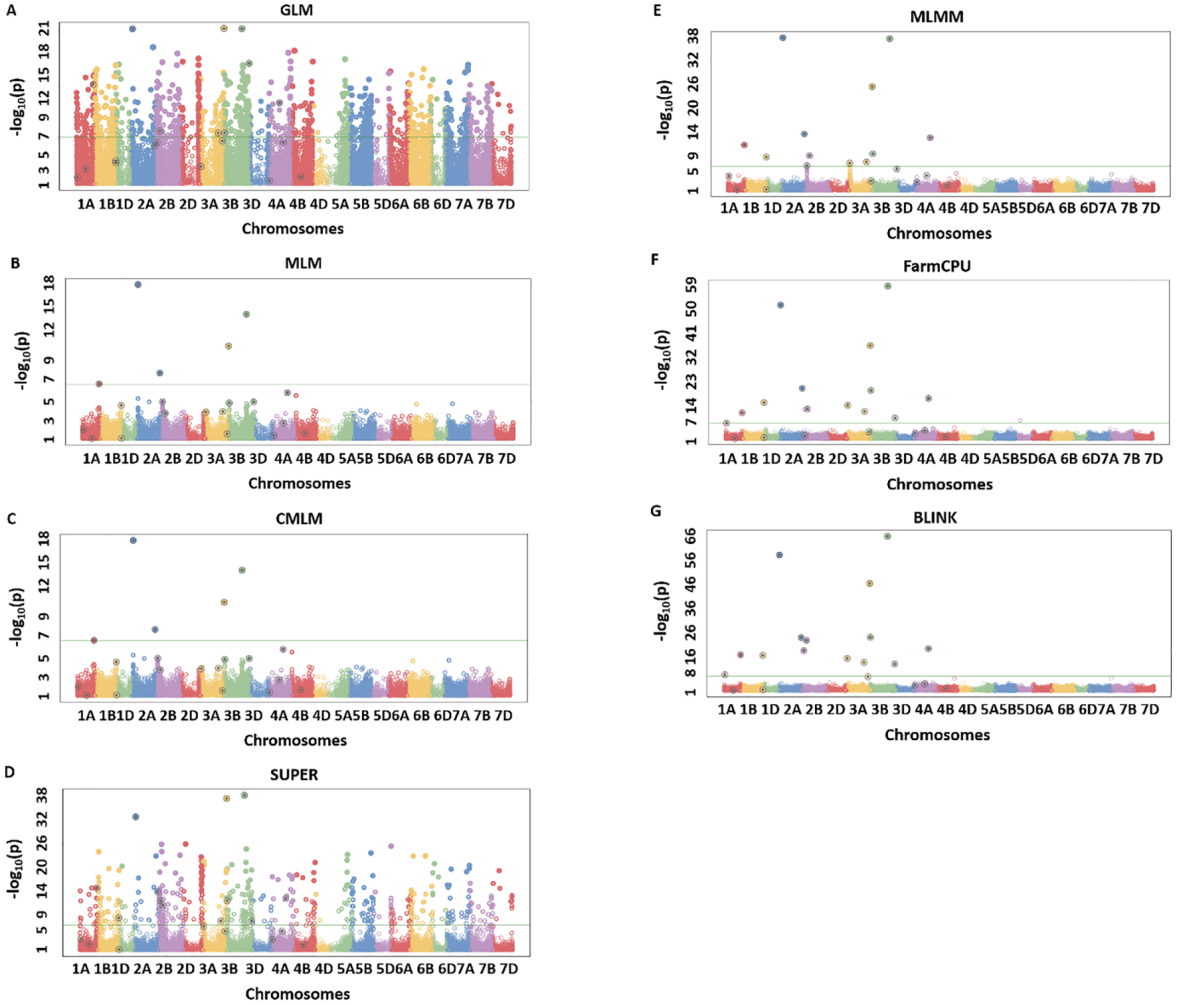
Manhattan plots for H80_Q20 simulated trait where quantitative trait loci (QTLs) had H = 80% and Q = 20 for seven different GWAS models including GLM **(A)**, MLM **(B)**, CMLM **(C)**, SUPER **(D)**, MLMM **(E)**, FarmCPU **(F)**, and BLINK **(G)**. The real QTLs are represented with the black circle over the SNP markers and Bonferroni correction of 0.05 was used for detecting the significant associations. The total number of real associations, false positives, and false negatives were assessed for each model to compare their performances.

**Figure 3:**
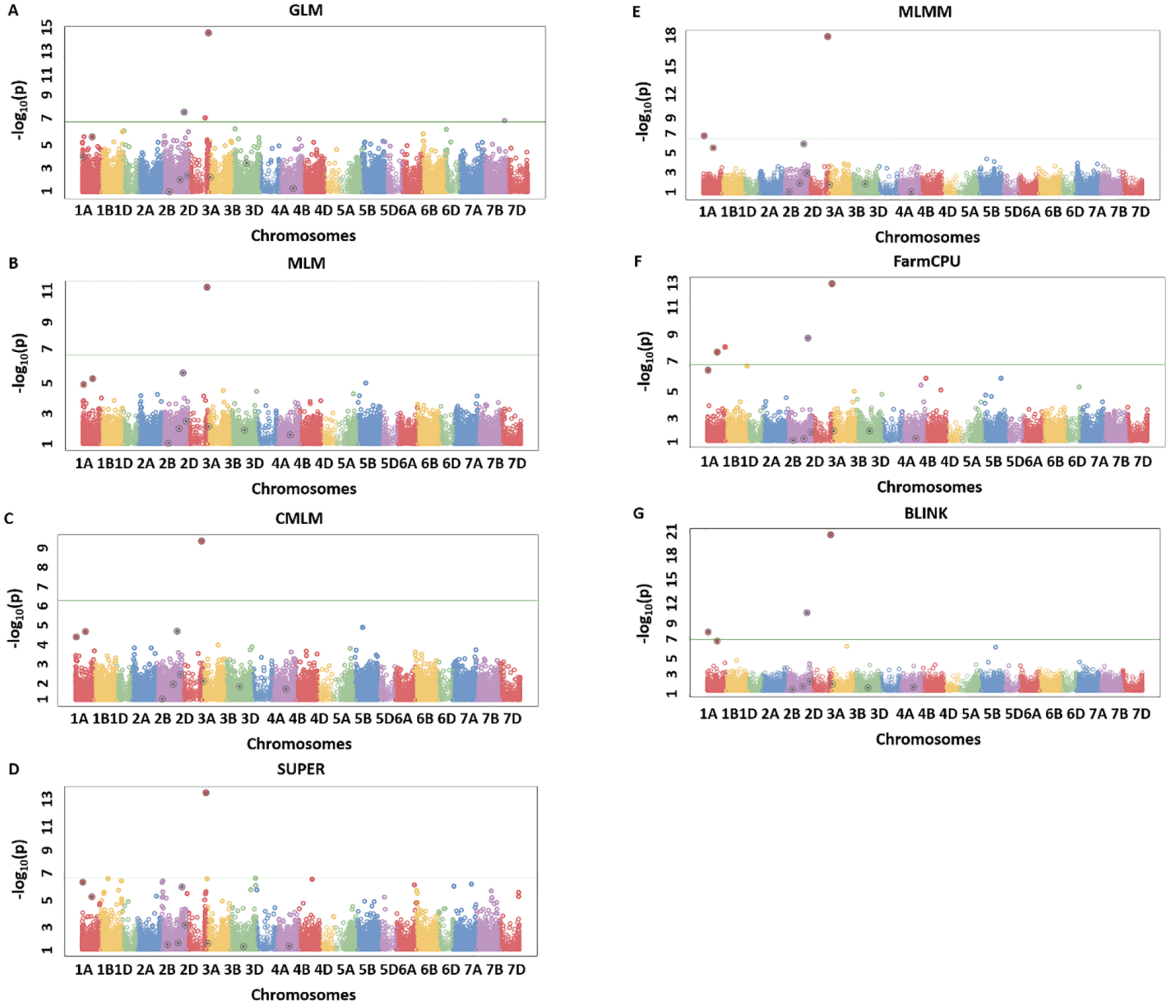
Manhattan plots for H20_Q10 simulated trait where quantitative trait loci (QTLs) had H = 20% and Q = 10 for seven different GWAS models including GLM **(A)**, MLM **(B)**, CMLM **(C)**, SUPER **(D)**, MLMM **(E)**, FarmCPU **(F)**, and BLINK **(G)**. The real QTLs are represented with the black circle over the SNP markers and Bonferroni correction of 0.05 was used for detecting the significant associations. The total number of real associations, false positives, and false negatives were assessed for each model to compare their performances.

The results from simulation studies were shown in the form of Manhattan plots to observe the true associations, false positives, and false negatives. We observed a similar trend in the model performance for all of the eight simulated traits, with BLINK being most reliable in regard to detecting true associations and controlling the false positives and false negatives. Due to space constraints, we provided results from the two extreme simulated traits namely H80_Q20 and H20_Q10 (**Figures 2**, **3**) (**Table 2**), but the same trends were observed for the remaining simulated traits. For H80_Q20, we simulated models to have 20 QTLs, which can explain 80% of the genetic variation. The GLM and SUPER models performed worse giving many false positives as evident from **Figures 2A, D**. Moreover, MLM and CMLM performed similarly by detecting five out of the 20 real QTLs, thus giving us a large number of false negatives, suggesting overfitting of the model (**Figures 2B, C**). MLMM was able to detect 11 of the 20 QTLs, but the number of false negatives was still high (**Figure 2E**). FarmCPU reduced the number of false negatives compared to MLMM by detecting 13 QTLs, but this model produced a couple of false positives (**Figure 2F**). Finally, BLINK performed superior compared to the above six models by detecting 15 of the 20 simulated QTLS with no false positives (**Figure 2G**). These results suggest that BLINK should be used if a trait has high heritability and controlled by a large number of QTLs.

A similar trend was observed for the H20_Q10 simulated trait, with the amount of genetic variation controlled being comparatively less than the H80 simulations. We observed that none of the models were able to detect all the simulated QTLs, but BLINK still performed better than all other models. GLM was able to detect two true associations with two false positives (**Figure 3A**). MLM, CMLMM, and SUPER were only able to detect one true association out of the 10 simulated QTLs (**Figures 3B–D**). MLMM performed better than the above four models by detecting two real associations and avoiding false positives (**Figure 3E**). Even though FarmCPU was able to detect three real associations, it produced one false positive (**Figure 3F**). Finally, BLINK was able to detect four real associations with complete avoidance of false positives (**Figure 3G**). These results were consistent with H80_Q20, as BLINK performed best under both scenarios, and Q-Q plots also clearly demonstrated the superiority of this model. These two analyses validated that BLINK should be used for mapping studies in the NAM population.

### Multiple threshold methods for significant associations

Multiple comparison methods, namely, Bonferroni correction, FDR, and positive FDR, were compared for significant association with a cutoff *P*-value of 0.05 for all methods. We performed the multiple comparisons for all simulated traits but for brevity, results for the H80_Q20 simulated trait is provided (**Table 3**). However, the results for all other simulated traits were similar (results not shown). We compared the number of significant associations identified after multiple comparison adjustments. In the case of GLM and SUPER, even after multiple comparison adjustments, there were a large number of false positives, suggesting the loose fitting of these models (**Table 3**). Similar results were observed from the Q-Q plot of GLM and SUPER, where a large number of false positives were evident (**Figures 1A, D**). Complex models, namely MLM, CMLM, MLMM, and FarmCPU were able to control the false positives after performing multiple comparison adjustments, especially using the Bonferroni correction (**Table 3**). However, there were false negatives for these complex models due to over-conservative nature of the Bonferroni threshold. In the case of BLINK, we were able to identify 15 and 18 significant associations with the Bonferroni correction and FDR, respectively. These results suggest that multiple comparison methods should be employed for controlling false positives and false negatives, in addition to the different statistical models.

**Table 3:** Total significant association detected for H80_Q20 simulated trait using four multiple threshold comparison methods, namely Bonferroni correction, FDR, PFDR, and −Log_10_ *P* > 3.5 for all seven models evaluated in this study.

Model	Bonferroni correction	False discovery rate (FDR)	Positive false discovery rate (PFDR)	−Log_10_ *P* > 3.5
GLM	3159	11,629	14,623	9102
MLM	8	9	9	263
CMLM	8	9	9	263
SUPER	868	1992	2531	6732
MLMM	12	52	68	1967
FarmCPU	15	33	46	2009
BLINK	15	18	18	1946

### Marker trait associations in the NAM population

**Figure 4:**
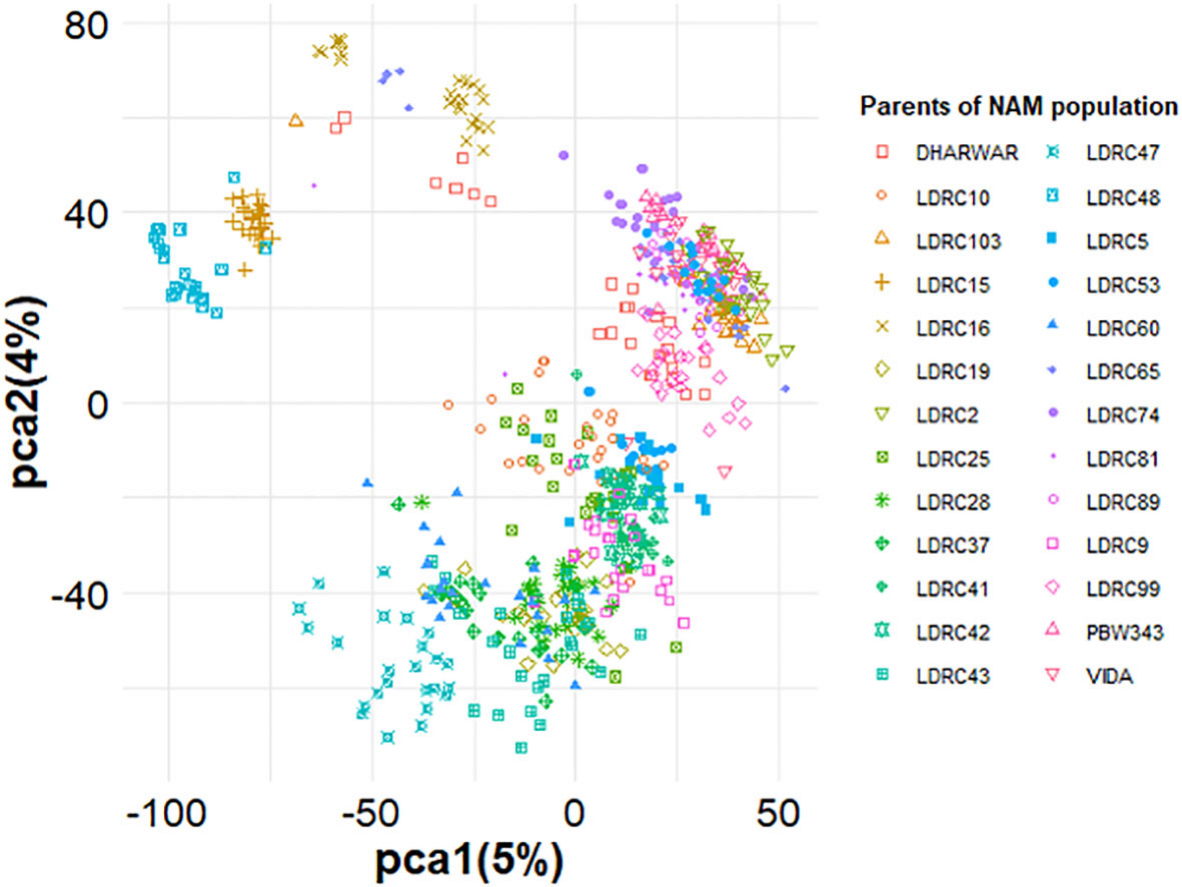
Principal components analysis of the NAM population containing 26 diverse founder parents. The absence of clear distinction among subpopulations is due to the use of common parents for obtaining the population. All the 26 founder parents’ families are represented for the reference.

PCA showed the presence of two main subgroups in the NAM, but the distinction among the different RILs was not clear (**Figure 4**). Furthermore, the first PC1 explained only 5% of the variation, while the second PC2 explained only 4% of the total variation in the population (**Figure 4**). The first two PCs were included as the covariate in the GWAS models to adjust for small subgroups observed in PCA.

Q-Q plots were assessed for these 11 phenotypic traits, and we observed that BLINK performed the best for controlling the false positives and false negatives (results not shown). Even the simulated studies suggested that BLINK should be used for the NAM population of wheat (**Figures 1**–**3**). Hence, MTAs for all phenotypic traits were performed using the BLINK model. We observed 45 MTAs for these traits, and the majority of these were distributed over the A and B genomes of wheat (**Table 4**). We identified three MTAs for plant height, which cumulatively explained 39.6% of the total variation and were located on chromosome 4B and 6B. In the case of days to heading and test weight, there were four and five significant MTAs, which cumulatively explained 16.9% and 17.1% of the total variation, respectively (**Table 4**). Furthermore, one, three, three, eight, nine, and one significant MTAs were obtained for NDVI, NWI, GNDVI, PRI, NCPI, and ARI, that cumulatively explain the 6.2%, 11.9%, 12.2%, 49.0%, 39.2%, and 2.2% of the total variations, respectively (**Table 4**). The chromosomal region 6A (CAP7_c4283_67) was shared among the NDVI and GNDVI indices. Similarly, chromosomal region 3A (SpringWheatNAM_tag_83405:72) and 7A (SpringWheatNAM_tag_104471) were shared between NWI and NCPI, and PRI and NCPI, respectively.

**Table 4:** Marker trait association for the eleven phenotypic traits obtained using the BLINK model for the NAM population of spring wheat planted for three years (2014–2016) in the U.S. Pacific Northwest.

Trait	Sr. No.	SNP name	Chromosome	Position on chromosome (cM)	Alleles	Minor allele frequency	R^2^	*P*-value
Plant height	1	IAAV971	4B	40752468	A/T	0.47	31.4	9.75E-37
	2	Excalibur_c17206_329	4B	573275528	C/T	0.12	2.2	5.81E-12
	3	SpringWheatNAM_tag_77531	6B	715850864	A/G	0.49	6.0	1.37E-07
Days to heading	1	RAC875_c9833_297	1A	394260203	A/C	0.16	5.6	5.02E-08
	2	SpringWheatNAM_tag_1669:12	1B	420423385	C/T	0.31	7.9	2.13E-07
	3	SpringWheatNAM_tag_206792	6A	122815244	A/T	0.47	2.3	1.36E-07
	4	SpringWheatNAM_tag_119869	7D	58770625	G/T	0.13	1.04	1.22E-07
Test weight	1	SpringWheatNAM_tag_312652	1D	432302805	T/C	0.32	0.21	1.26E-06
	2	SpringWheatNAM_tag_178261:78	2B	545760724	C/T	0.16	4.36	1.16E-06
	3	SpringWheatNAM_tag_54608	2D	20750397	C/T	0.24	4.00	3.80E-07
	4	SpringWheatNAM_tag_30111:80	4B	637388295	C/G	0.20	4.30	3.10E-10
	5	SpringWheatNAM_tag_73760:49	5B	586876059	G/C	0.21	4.2	1.21E-08
NDVI	1	CAP7_c4283_67	6A	581746429	T/G	0.14	6.21	1.38E-07
NWI	1	RAC875_rep_c112916_263	2B	762708836	T/A	0.32	4.89	2.56E-07
	2	SpringWheatNAM_tag_176235	2D	11052027	C/A	0.49	0.01	2.46E-07
	3	SpringWheatNAM_tag_83405:72	3A	55486364	A/G	0.33	6.96	1.77E-08
GNDVI	1	SpringWheatNAM_tag_284432	2B	49546799	G/T	0.13	0.45	7.59E-07
	2	Ku_c2936_1987	2B	782154354	G/A	0.15	5.83	1.55E-07
	3	CAP7_c4283_67	6A	581746429	T/G	0.14	5.95	2.05E-07
PRI	1	BS00087757_51	3B	6250439	A/C	0.36	11.69	1.51E-26
	2	SpringWheatNAM_tag_81419	4B	480696216	A/T	0.46	0.067	9.19E-08
	3	SpringWheatNAM_tag_150054:75	5B	476890298	T/C	0.11	12.66	4.96E-08
	4	SpringWheatNAM_tag_99841	5B	702607859	T/G	0.28	1.98	4.45E-07
	5	SpringWheatNAM_tag_110769	6B	615640864	G/C	0.19	11.93	8.67E-07
	6	RAC875_c13216_111	6B	645353741	C/G	0.20	1.87	2.93E-07
	7	SpringWheatNAM_tag_11038	7A	12697901	C/T	0.28	3.07	4.04E-07
	8	SpringWheatNAM_tag_104471	7A	620164337	C/A	0.25	5.74	2.47E-07
NCPI	1	Tdurum_contig54784_485	2B	718967996	A/G	0.23	8.94	2.76E-08
	2	SpringWheatNAM_tag_83405:72	3A	55486364	A/G	0.33	7.90	4.24E-07
	3	SpringWheatNAM_tag_7460	3B	340216648	T/C	0.37	0.76	9.74E-07
	4	SpringWheatNAM_tag_93215	3B	655303120	G/T	0.29	0.88	6.13E-08
	5	SpringWheatNAM_tag_1115	4A	672100114	G/A	0.13	0.87	4.55E-07
	6	SpringWheatNAM_tag_73855:52	4B	36455135	C/T	0.25	7.52	7.58E-09
	7	Excalibur_rep_c109299_159	6B	47849285	A/C	0.11	4.40	5.87E-07
	8	SpringWheatNAM_tag_21926:27	6B	82543505	G/A	0.39	2.34	7.46E-07
	9	SpringWheatNAM_tag_104471	7A	620164337	C/A	0.25	5.59	1.11E-06
ARI	1	SpringWheatNAM_tag_233767	5B	412271965	T/C	0.12	2.16	2.46E-06
Grain protein content	1	wsnp_Ex_c23795_33033150	5A	679665943	T/G	0.14	5.10	7.96E-08
	2	SpringWheatNAM_tag_30378:21	5B	397964122	A/G	0.16	0.53	4.50E-07
	3	Kukri_c31995_1948	6D	402127283	A/T	0.47	2.81	6.32E-08
	4	SpringWheatNAM_tag_37052	7A	712814970	G/A	0.16	4.04	6.55E-08
	5	SpringWheatNAM_tag_103903	7B	437172173	G/C	0.31	0.51	8.41E-09
	6	SpringWheatNAM_tag_78736	7B	449883322	C/A	0.28	6.11	5.06E-08
Grain yield	1	SpringWheatNAM_tag_65895	1B	237437644	A/T	0.36	4.31	8.79E-08
	2	IAAV971	4B	40752468	A/T	0.46	12.33	4.10E-13

### Model performance for height in wheat

**Figure 5:**
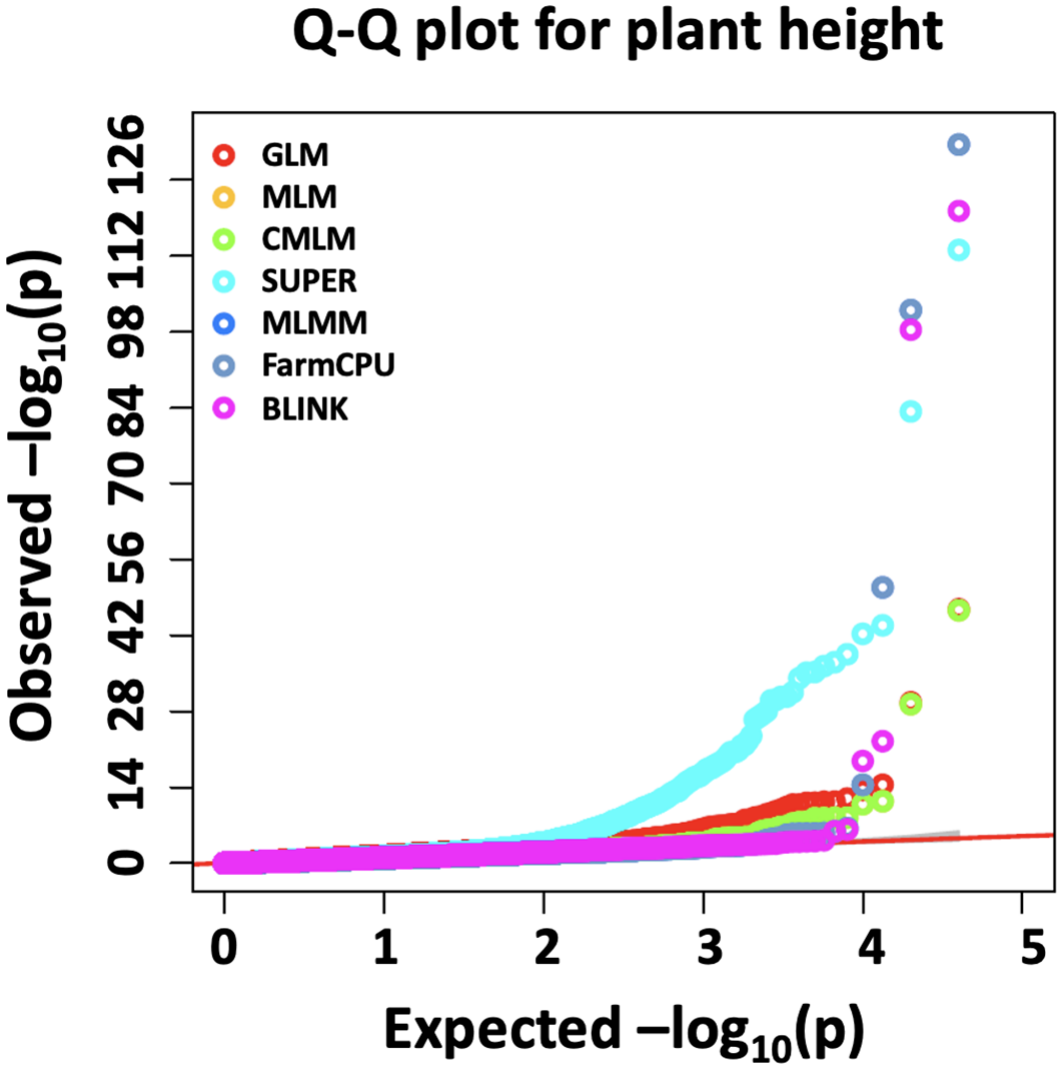
Comparison of Q-Q plots for seven different GWAS models, namely, GLM, MLM, CMLM, SUPER, MLMM, FarmCPU, and BLINK for marker trait associations for plant height in a NAM population of spring wheat.

**Figure 6:**
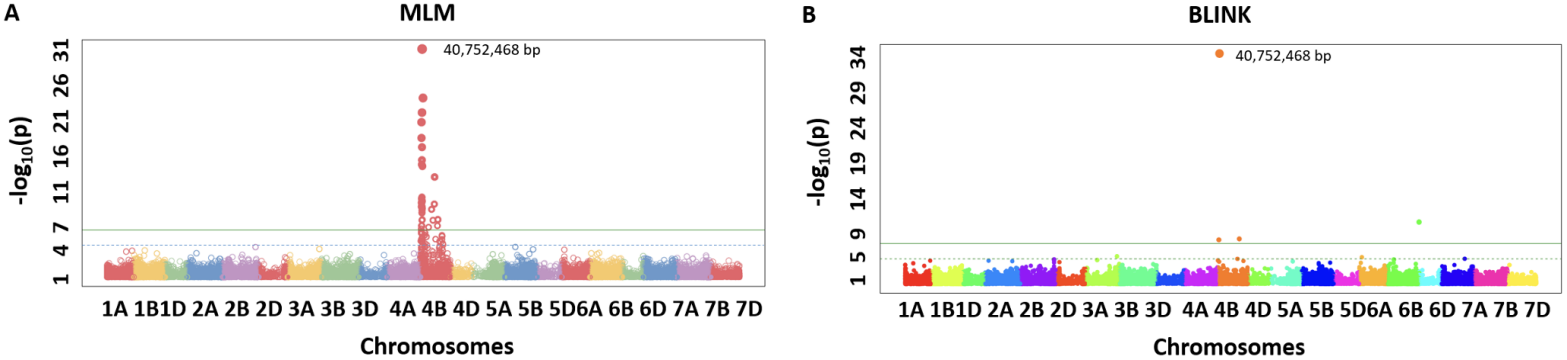
Manhattan plots for marker-trait associations for plant height using MLM **(A)** and BLINK **(B)** in the NAM population of spring wheat.

We identified the MTA of IAAV971 SNP marker with plant height that is located on 4BS at 40752468 bp. We validated the presence of *Rht1* genes (*Rh1-B1a/Rht1-B1b*) in these RILs with the use of kompetitive allele-specific polymorphic (KASP) markers (results not shown) (Grogan et al., 2016; Blake et al., 2019). Due to known position of the *Rht1* gene, plant height is the best trait in our population to compare the models which identify the best significant association. Q-Q plots were compared for all seven models used in this study and BLINK performed best regarding the control of false positives (**Figure 5**). We also showed the MTAs for plant height with two models, namely, MLM and BLINK. BLINK was able to separate a clear association with one SNP marker compared to MLM that provided many MTAs on 4B (**Figure 6**). Even though the marker having the highest significant threshold was the same for both models, BLINK was able to control all the linked markers by using its linkage-based mapping criteria.

## Discussion

### The best statistical model for MTAs in the NAM population

This study’s main aim was to compare seven different single and multi-locus models for MTAs in a NAM population of wheat for simulated and complex quantitative traits. The GLM resulted in a large deviation of the Q-Q plot from the 1:1 line (**Figure 1**) and a large number of false positives as evident from Manhattan plots (**Figures 2**, **3**), thus indicating this model is not appropriate for MTAs in this NAM population of wheat. These results are similar to previous studies conducted in humans, maize, and Arabidopsis, thus validating that GLM model is not appropriate for mapping complex quantitative traits (Huang et al., 2018; Liu et al., 2016; Zhang et al., 2010). The large number of false positives in GLM is due to ignoring relatedness among the individuals, which are not completely accounted for by population structure parameters (Zhang et al., 2010).

Complex GWAS models, namely, MLM, CMLM, MLMM, and FarmCPU, efficiently control for false positives but comparatively result in several false negatives in simulated studies, except when the trait has a low heritability (**Figures 2**, **3**). These false negatives were produced due to overfitting in these models (Huang et al., 2018). The MLM and CMLM usually result in false negatives for complex traits, and these results were validated in previous studies (Liu et al., 2016; Kaler et al., 2020). This is because these models test a single marker at a time, thus completely ignoring the genetic makeup of complex traits, which causes omission of other small effect associations (Segura et al., 2012). MLMM tests multiple QTNs as a cofactor in the stepwise regression MLM, using heritable variance criteria for forward inclusion and backward elimination of QTNs. However, this model is shown to perform superior for large effect QTLs and thus produces false negatives, especially for the small effect QTLs in our study. Similar results were previously reported from other studies (Wen et al., 2018; Liu et al., 2016; Kaler et al., 2020).

FarmCPU performed superior to all other models except BLINK; this is because FarmCPU divides the MLM into a fixed and random effect model. The fixed effect tests multiple markers at a time, and the random effect model selected the associated markers to obtain kinship. These two processes are performed iteratively and control the false positives in the model (Liu et al., 2016). In this study, the recently developed BLINK model performed superior to detect real associations with control of false positives and false negatives for the simulation experiment. This model was better than the FarmCPU model, as FarmCPU assumes that QTN bins are evenly distributed over the genome, completely ignoring linkage disequilibrium information. However, BLINK replaced the even distribution of QTN bins with linkage disequilibrium information (Huang et al., 2018).

### Multiple threshold methods comparison for significant associations

False positives and false negatives arise not only by selection of GWAS models but also due to less stringent or over conservative thresholds. Herein, we compared the results from four multiple threshold methods, namely, Bonferroni correction, FDR, PFRD, and −Log_10_ *P* > 3.5 under simulation studies from all the seven GWAS models (**Table 2**). Our results suggest that significant threshold methods play an equally important role in controlling spurious associations, in addition to the GWAS models. Bonferroni correction was observed to be a superior threshold method in the simulation studies for the majority of the GWAS models used. Even though Bonferroni correction was not able to reduce the number of false positives when used with GLM and MLMM, it did perform efficiently for all other models (**Table 2**).

The number of false negatives observed for complex models such as MLM, CMLM, MLMM, and FarmCPU with Bonferroni correction of 0.05 was not due to this threshold, but it was due to the GWAS model’s performance (**Figures 2**, **3**). Manhattan plots from simulation studies for MLM, CMLM, MLMM, and FarmCPU show that false negatives were not due to Bonferroni threshold, but due to very low cutoff *p*-values, and suggests the inefficiency of the associated models. Furthermore, Bonferroni correction, FDR, and PFDR performed similarly for the BLINK model with complete avoidance of false negatives and false positives (**Table 3**). These results suggest that the utilization of an efficient GWAS model should be complemented with the best significant threshold method for obtaining true associations within breeding programs. The probable reason for the same performance of all multi-comparison threshold methods in the case of BLINK is due to the working principle of the model. BLINK efficiently controls spurious associations with utilization of linkage disequilibrium present among the QTNs throughout the genome (Huang et al., 2018).

Several studies suggest that Bonferroni correction should be replaced with less stringent or novel thresholds such as FDR, PFDR, permutation test, Sidak correction, and Bayesian approaches (Hochberg, 1988; Holm, 1978; Benjamini and Hochberg, 1995; Šidák, 1967). This is due to assumptions of this method, which results in over-conservative significant threshold, obtained as the *P*-value/number of independent tests, and this does not consider that markers on the same chromosome could be independent (Hayes, 2013). However, in our simulation study, we observed that Bonferroni correction should be utilized over the FDR, PFDR, and random −log *P*-values as this method controls for both false positives and false negatives. Kaler and Purcell (2019) suggested that Bonferroni correction and FDR are highly conservative thresholds, and both methods result in a large number of false negatives. They developed a significant threshold using marker-based and broad-sense heritability, which was less conservative than the abovementioned thresholds in maize, soybean, and rice. However, our simulation results suggest that Bonferroni correction is the best regarding control of both false positives and false negatives in the BLINK model, and this should be used to report associations. Associations identified with Bonferroni thresholds help provide enough confidence that these markers can be used for marker-assisted selection, compared to less stringent methods, which require further validation.

### Marker trait associations for agronomic traits in the NAM population

A total of 20 MTAs were identified for five agronomic traits used in this study. Three loci were mapped on chromosomes 4B and 6B for plant height (**Table 4**). The most significant locus was mapped around the base position 40,752,468. This locus has a positive effect on plant height. The increase in plant height in the NAM population confirms that founder parents lack the *Rht-B1* gene (Blake et al., 2019). The locus mapped on 6B was previously mapped in several studies and has been validated that this locus is responsible for reducing the plant height in wheat (Malik et al., 2019; Zanke et al., 2014; Turuspekov et al., 2017). The same locus was also associated with yield due to height and lodging reduction.

Four loci were mapped on 1A, 1B, 6A, and 7D for regulating the days to heading in the population. The MTAs on chromosomes 1A, 1B, and 7D aid in reducing days to heading, whereas the remaining MTA has a positive effect on days to heading. The locus mapped on 7D was previously reported in a soft red winter wheat population and suggests that this locus controls heading time in wheat irrespective of wheat class and type (Gaire et al., 2020). Similarly, MTAs on 6A was previously reported to have a significant effect on heading date (Jamil et al., 2019). The QTL for grain yield mapped on chromosome 4B was also regulating height in this population (Peng et al., 1999), supporting the semi-dwarf wheat leads to higher grain yield. Furthermore, other MTAs mapped for grain protein content and grain yield were novel and not reported in the previous studies. The small effects of these gene regions suggest that genomic selection is the best alternative for predicting these complex traits in breeding programs (Bernardo, 2016).

### Marker trait associations for spectral reflectance indices in the NAM population

Spectral reflectance indices provide an indirect estimation of various physiological processes occurring in plants and could ultimately be used to select grain yield (Babar et al., 2006). In this study, 25 MTAs were associated with six SRIs, namely, NDVI, NWI, GNDVI, PRI, NCPI, and ARI. The loci contributing to SRIs are distributed across most of the chromosomes, suggesting widespread variation for these traits. NDVI and GNDVI provide indirect estimation about greenness, biomass content, chlorophyll concentration, and plant health (Gitelson et al., 1996; Prasad et al., 2007). NDVI and GNDVI measure reflection from the red and green region of the electromagnetic spectrum, thus providing information from different regions. We mapped a locus associated with marker CAP7_c4283_67 on chromosome 6A, which is associated with both NDVI and GNDVI. This locus has a negative effect on both indices and will ultimately aid in selecting against this locus in the breeding program. This locus was not identified in any other previous studies for mapping regions for NDVI and GNDVI (Prasad et al., 2007; Gizaw et al., 2018). The GDNVI locus mapped on 2B was previously mapped around the same position in wheat and has a positive effect on the trait (Gizaw et al., 2018).

PRI, NCPI, and ARI measure the reflection from the visible region of the electromagnetic spectrum and provides information about different pigments in the plants (Peñuelas et al., 1997). The location of these indices was different from other agronomic traits mapped in this study, and this will aid in the selection of these traits independently. The genomic region determined by marker SpringWheatNAM_tag_104471 on chromosome 7A was associated with PRI and NCPI in this study, suggesting the pleiotropic nature of this locus. The MTAs on chromosome 3B and 6B collocated with the previously mapped agronomic and yield-related traits in wheat. These are shown to have QTLs for grain filling duration, test weight, and spikelet number (Edae et al., 2014). NCPI and PRI are a good indicator of accessory pigments, chlorophyll concentration, and photosynthesis efficiency (Peñuelas et al., 1994). Hence, MTAs identified in this study will assist breeders in understanding how these regions can be used as proxy for selecting genotypes based on their physiological performance.

## Conclusion

This study compared the performances of seven different GWAS models on eight simulated and 11 real traits in a spring wheat NAM population. The BLINK model was observed to be best for association mapping in wheat, as observed from the Q-Q plots and simulated QTLs identified by the GWAS models. This model performed best for controlling false positives and false negatives under all the genetic architecture of the simulated traits. We observed that multiple threshold methods complement GWAS models for controlling spurious associations, with the Bonferroni correction observed to be best for controlling false positives and false negatives under simulation studies. We concluded that the BLINK model should be used for association mapping in wheat with the Bonferroni correction as the significant threshold method. Markers trait association for 11 different agronomic and spectral traits identified 45 significant associations using a Bonferroni correction of 0.05 with the BLINK model. As all the founder parents of the NAM population were landraces, this study identified various novel associations.

## Data Availability

The datasets presented in this study can be found in online repositories. The names of the repository/repositories and accession number(s) can be found in the article/supplementary material.

## References

[B1] Babar M. A. Reynolds M. P. Van Ginkel M. Klatt A. R. Raun W. R. Stone M. L. (2006). Spectral reflectance to estimate genetic variation for in-season biomass, leaf chlorophyll, and canopy temperature in wheat. Crop Sci. 46, 1046–1057. doi: 10.2135/cropsci2005.0211

[B2] Benjamini Y. Hochberg Y. (1995). Controlling the false discovery rate: A practical and powerful approach to multiple testing. J. R. Stat. Society: Ser. B (Methodological) 57, 289–3005. doi: 10.1111/j.2517-6161.1995.tb02031.x

[B3] Bernardo R. (2008). Molecular markers and selection for complex traits in plants: learning from the last 20 years. Crop Sci. 48, 1649–1664. doi: 10.2135/cropsci2008.03.0131

[B4] Bernardo R. (2016). Bandwagons I, too, have known. Theor. Appl. Genet. 129, 2323–2332. doi: 10.1007/s00122-016-2772-5 27681088

[B5] Blake N. K. Pumphrey M. Glover K. Chao S. Jordan K. Jannick J. L.. (2019). Registration of the triticeae-cap spring wheat nested association mapping population. J. Plant Registrations 13, 294–297. doi: 10.3198/jpr2018.07.0052crmp

[B6] Cullis B. R. Smith A. B. Coombes N. E. (2006). On the design of early generation variety trials with correlated data. J. Agricultural Biological Environ. Stat 11, 381–935. doi: 10.1198/108571106X154443

[B7] Dudbridge F. Gusnanto A. (2008). Estimation of significance thresholds for genomewide association scans. Genet. Epidemiol. 32, 227–345. doi: 10.1002/gepi.20297 18300295 PMC2573032

[B8] Edae E. A. Byrne P. F. Haley S. D. Lopes M. S. Reynolds M. P. (2014). Genome-wide association mapping of yield and yield components of spring wheat under contrasting moisture regimes. Theor. Appl. Genet. 127, 791–8075. doi: 10.1007/s00122-013-2257-8 24408378

[B9] Gaire R. Ohm H. Brown-Guedira G. Mohammadi M. (2020). Identification of regions under selection and loci controlling agronomic traits in a soft red winter wheat population. Plant Genome 13, e20031. doi: 10.1002/tpg2.20031 33016613 PMC12807307

[B10] Gitelson A. A. Kaufman Y. J. Merzlyak M. N. (1996). Use of a green channel in remote sensing of global vegetation from EOS- MODIS. Remote Sens. Environ. 58, 289–985. doi: 10.1016/S0034-4257(96)00072-7

[B11] Gizaw S. A. Godoy J. G. V. Garland-Campbell K. Carter A. H. (2018). Using spectral reflectance indices as proxy phenotypes for genome-wide association studies of yield and yield stability in Pacific Northwest Winter wheat. Crop Sci. 58, 1232–1415. doi: 10.2135/cropsci2017.11.0710

[B12] Grogan S. M. Brown-Guedira G. Haley S. D. McMaster G. S. Reid S. D. Smith J.. (2016). Allelic variation in developmental genes and effects on winter wheat heading date in the U.S. Great Plains. PLoS ONE 11 (4), e0152852. doi: 10.1371/journal.pone.0152852 27058239 PMC4825937

[B13] Hayes B. (2013). Overview of statistical methods for genome-wide association studies (GWAS) (Totowa, NJ: Humana Press), 149–169. doi: 10.1007/978-1-62703-447-0_6 23756890

[B14] Hochberg Y. (1988). A sharper bonferroni procedure for multiple tests of significance. Biometrika 75, 800–802. doi: 10.1093/biomet/75.4.800

[B15] Holm S. (1978). A simple sequentially rejective multiple test procedure. Scandinavian J. Stat 6, 65–70.

[B16] Huang M. Liu X. Zhou Y. Summers R. M. Zhang Z. (2018). BLINK: A package for the next level of genome-wide association studies with both individuals and markers in the millions. GigaScience 8, 1–125. doi: 10.1093/gigascience/giy154 PMC6365300 30535326

[B17] Jamil M. Ali A. Gul A. Ghafoor A. Napar A. A. Ibrahim A. M. H.. (2019). Genome-wide association studies of seven agronomic traits under two sowing conditions in bread wheat. BMC Plant Biol. 19, 1–185. doi: 10.1186/s12870-019-1754-6 31003597 PMC6475106

[B18] Johnson R. C. Nelson G. W. Troyer J. L. Lautenberger J. A. Kessing B. D. Winkler C. A.. (2010). Accounting for multiple comparisons in a genome-wide association study (GWAS). BMC Genomics 11, 1–65. doi: 10.1186/1471-2164-11-724 21176216 PMC3023815

[B19] Jordan K. W. Wang S. He F. Chao S. Lun Y. Paux E.. (2018). The genetic architecture of genome-wide recombination rate variation in allopolyploid wheat revealed by nested association mapping. Plant J. 95, 1039–1054. doi: 10.1111/tpj.14009 29952048 PMC6174997

[B20] Kaler A. S. Gillman J. D. Beissinger T. Purcell L. C. (2020). Comparing different statistical models and multiple testing corrections for association mapping in soybean and maize. Front. Plant Sci. 10. doi: 10.3389/fpls.2019.01794 PMC7052329 32158452

[B21] Kaler A. S. Purcell L. C. (2019). Estimation of a significance threshold for genome-wide association studies. BMC Genomics 20, 1–85. doi: 10.1186/s12864-019-5992-7 31357925 PMC6664749

[B22] Kaur B. Sandhu K. S. Kamal R. Kaur K. Singh J. Röder M. S.. (2021). Omics for the improvement of abiotic, biotic and agronomic traits in major cereals: applications, challenges, and prospects. Plants 10, 1989. Available at: https://phytozome-next.jgi.doe.gov/. 34685799 10.3390/plants10101989 PMC8541486

[B23] Lander E. S. Botstein D. (1989). Mapping mendelian factors underlying quantitative traits using RFLP linkage maps. Genetics 121, (1). doi: 10.1093/genetics/121.1.185 2563713 PMC1203601

[B24] Li H. Bradbury P. Ersoz E. Buckler E. S. Wang J. (2011). Joint QTL linkage mapping for multiple-cross mating design sharing one common parent. PLoS One 6, e17573. doi: 10.1371/journal.pone.0017573 21423655 PMC3057965

[B25] Li H. Hearne S. Bänziger M. Li Z. Wang J. (2010). Statistical properties of QTL linkage mapping in biparental genetic populations. Heredity 105, 257–267. doi: 10.1038/hdy.2010.56 20461101

[B26] Lipka A. E. Tian F. Wang Q. Peiffer J. Li M. Bradbury P. J.. (2012). GAPIT: genome association and prediction integrated tool. Bioinformatics 28, 2397–2995. doi: 10.1093/bioinformatics/bts444 22796960

[B27] Liu X. Huang M. Fan B. Buckler E. S. Zhang Z. (2016). Iterative usage of fixed and random effect models for powerful and efficient genome-wide association studies. PLoS Genet. 12, e10057675. doi: 10.1371/journal.pgen.1005767 PMC4734661 26828793

[B28] Malik P. L. Janss L. Nielsen L. K. Borum F. Jørgensen K. Eriksen B.. (2019). Breeding for dual-purpose wheat varieties using marker–trait associations for biomass yield and quality traits. Theor. Appl. Genet. 132, 3375–3985. doi: 10.1007/s00122-019-03431-z 31555887

[B29] McMullen M. D. Kresovich S. Villeda H. S. Bradbury P. Li H. Sun Q.. (2009a). Supporting online material for: genetic properties of the maize nested association mapping population. Science 325, 737–741. doi: 10.1126/science.1174320 19661427

[B30] McMullen M. D. Kresovich S. Villeda H. S. Bradbury P. Li H. Sun Q.. (2009b). Genetic properties of the maize nested association mapping population. Science 325, 737–740. doi: 10.1126/science.1174320 19661427

[B31] Nice L. M. Steffenson B. J. Blake T. K. Horsley R. D. Smith K. P. Muehlbauer G. J.. (2017). Mapping agronomic traits in a wild barley advanced backcross – nested association mapping population. Crop Sci. 57, 1199–12105. doi: 10.2135/cropsci2016.10.0850

[B32] Pe’er I. Yelensky R. Altshuler D. Daly M. J. (2008). Estimation of the multiple testing burden for genomewide association studies of nearly all common variants. Genet. Epidemiol. 32, 381–855. doi: 10.1002/gepi.20303 18348202

[B33] Peng J. Richards D. E. Hartley N. M. Murphy G. P. Devos K. M. Flintham J. E.. (1999). [amp]]lsquo;Green revolution’ Genes encode mutant gibberellin response modulators. Nature 400, 256–261. doi: 10.1038/22307 10421366

[B34] Peñuelas J. Gamon J. A. Fredeen A. L. Merino J. Field C. B. (1994). Reflectance indices associated with physiological changes in nitrogen- and water-limited sunflower leaves. Remote Sens. Environ. 48, 135–146. doi: 10.1016/0034-4257(94)90136-8

[B35] Peñuelas J. Llusia J. Piñol J. Filella I. (1997). Photochemical reflectance index and leaf photosynthetic radiation-use efficiency assessment in mediterranean trees. Int. J. Remote Sens 18, 2863–2868. doi: 10.1080/014311697217387

[B36] Poland J. A. Brown P. J. Sorrells M. E. Jannink J. L. (2012). Development of high-density genetic maps for barley and wheat using a novel two-enzyme genotyping-by-sequencing approach. PLoS One 7, e32253. doi: 10.1371/journal.pone.0032253 22389690 PMC3289635

[B37] Prasad B. Carver B. F. Stone M. L. Babar M. A. Raun W. R. Klatt A. R. (2007). Genetic analysis of indirect selection for winter wheat grain yield using spectral refl ectance indices. Crop Sci. 47, 1416–1425. doi: 10.2135/cropsci2006.08.0546

[B38] Price A. L. Patterson N. J. Plenge R. M. Weinblatt M. E. Shadick N. A. Reich D. (2006). Principal components analysis corrects for stratification in genome-wide association studies. Nat. Genet. 38, 904–909. doi: 10.1038/ng1847 16862161

[B39] Pritchard J. K. Stephens M. Donnelly P. (2000). Inference of Population Structure Using MultI locus GeNotyPe DatA. Genetics 155, 945–959. doi: 10.1093/genetics/155.2.945 PMC1461096 10835412

[B40] R Core Team (2020). A Language and Environment for Statistical Computing (Vienna, Austria: R Foundation for Statistical Computing). Available at: https://Www.R-Project.Org/.

[B41] R Development Core Team (2020). R: A Language and Environment for Statistical Computing (Vienna, Austria: R Foundation for Statistical Computing), 201. Available at: http://www.r-project.org, ISBN: 3-900.

[B42] Rodríguez F. Alvarado G. Pacheco Á. Burgueño J. (2018). ACBD-R. Augmented Complete Block Design with R for Windows. Version 4.0. Texcoco de Mora, Mexico: CIMMYT Research Data & Software Repository Network.

[B43] Sandhu K. S. Lozada D. N. Zhang Z. Pumphrey M. O. Carter A. H. (2021b). Deep learning for predicting complex traits in spring wheat breeding program. Front. Plant Sci. 11. doi: 10.3389/fpls.2020.613325 PMC7813801 33469463

[B44] Sandhu K. S. Mihalyov P. D. Lewien M. J. Pumphrey M. O. Carter A. H. (2021c). Combining genomic and phenomic information for predicting grain protein content and grain yield in spring wheat. Front. Plant Sci. 12. doi: 10.3389/fpls.2021.613300 PMC7907601 33643347

[B45] Sandhu K. S. Mihalyov P. D. Lewien M. J. Pumphrey M. O. Carter A. H. (2021d). Genome-wide association studies and genomic selection for grain protein content stability in a nested association mapping population of spring wheat. Agronomy 11, 2528. doi: 10.3390/agronomy11122528

[B46] Sandhu K. Patil S. S. Pumphrey M. Carter A. (2021a). Multitrait machine- and deep-learning models for genomic selection using spectral information in a wheat breeding program. Plant Genome 14, e20119. doi: 10.1002/TPG2.20119 34482627

[B47] SAS Institute Inc (2011). SAS® 9.3 System Options: Reference. Cary, North Carolina: SAS Institute.

[B48] Segura V. Vilhjálmsson B. J. Platt A. Korte A. Seren Ü. Long Q.. (2012). An efficient multi-locus mixed-model approach for genome-wide association studies in structured populations. Nat. Genet. 44, 825–305. doi: 10.1038/ng.2314 22706313 PMC3386481

[B49] Šidák Z. (1967). Rectangular confidence regions for the means of multivariate normal distributions. J. Am. Stat. Assoc. 62, 626–633. doi: 10.1080/01621459.1967.10482935

[B50] Singh J. Chhabra B. Raza A. Yang S. H. Sandhu K. S. (2023). Important wheat diseases in the US and their management in the 21st century. Front. Plant Sci. 13. doi: 10.3389/FPLS.2022.1010191/BIBTEX PMC9877539 36714765

[B51] Song Q. Yan L. Quigley C. Jordan B. D. Fickus E. Schroeder S.. (2017). Genetic characterization of the soybean nested association mapping population. Plant Genome 10, plantgenome2016.10.010. doi: 10.3835/plantgenome2016.10.0109 28724064

[B52] Stich B. Möhring J. Piepho H. P. Heckenberger M. Buckler E. S. Melchinger A. E. (2008). Comparison of mixed-model approaches for association mapping. Genetics 178, 1745–1545. doi: 10.1534/genetics.107.079707 18245847 PMC2278052

[B53] Turuspekov Y. Baibulatova A. Yermekbayev K. Tokhetova L. Chudinov V. Sereda G.. (2017). GWAS for plant growth stages and yield components in spring wheat (Triticum aestivum L.) harvested in three regions of Kazakhstan. BMC Plant Biol. 17, 1–115. doi: 10.1186/s12870-017-1131-2 29143598 PMC5688510

[B54] VanRaden P. M. (2008). Efficient methods to compute genomic predictions. J. Dairy Sci. 91, 4414–4423. doi: 10.3168/jds.2007-0980 18946147

[B55] Wang D. Sun Y. Stang P. Berlin J. A. Wilcox M. A. Li Q. (2009). Comparison of methods for correcting population stratification in a genome-wide association study of rheumatoid arthritis: principal-component analysis versus multidimensional scaling. BMC Proc. 3, 109. doi: 10.1186/1753-6561-3-s7-s109 PMC2795880 20017973

[B56] Wang Q. Tian F. Pan Y. Buckler E. S. Zhang Z. (2014). A SUPER powerful method for genome wide association study. PLoS One 9, e1076845. doi: 10.1371/journal.pone.0107684 PMC4172578 25247812

[B57] Wang S. Wong D. Forrest K. Allen A. Chao S. Huang B. E.. (2014). Characterization of polyploid wheat genomic diversity using a high-density 90 000 single nucleotide polymorphism array. Plant Biotechnol. J. 12, 787–796. doi: 10.1111/pbi.12183 24646323 PMC4265271

[B58] Wen Y. J. Zhang H. Ni Y. L. Huang B. Zhang J. Feng J. Y.. (2018). Methodological implementation of mixed linear models in multi-locus genome-wide association studies. Briefings Bioinf. 19, 700–7125. doi: 10.1093/bib/bbw145 PMC6054291 28158525

[B59] Wu C. DeWan A. Hoh J. Wang Z. (2011). A comparison of association methods correcting for population stratification in case-control studies. Ann. Hum. Genet. 75, 418–275. doi: 10.1111/j.1469-1809.2010.00639.x 21281271 PMC3215268

[B60] Würschum T. Liu W. Gowda M. Maurer H. P. Fischer S. Schechert A.. (2012). Comparison of biometrical models for joint linkage association mapping. Heredity 108, 332–340. doi: 10.1038/hdy.2011.78 21878984 PMC3282402

[B61] Yu J. Holland J. B. Mcmullen M. D. Buckler E. S. (2008). Genetic design and statistical power of nested association mapping in maize. Genetics 178, 539–551. doi: 10.1534/genetics.107.074245 18202393 PMC2206100

[B62] Yu J. Pressoir G. Briggs W. H. Bi I. V. Yamasaki M. Doebley J. F.. (2006). A unified mixed-model method for association mapping that accounts for multiple levels of relatedness. Nat. Genet. 38, 203–208. doi: 10.1038/ng1702 16380716

[B63] Zanke C. D. Ling J. Plieske J. Kollers S. Ebmeyer E. Korzun V.. (2014). Whole genome association mapping of plant height in winter wheat (Triticum aestivum L.). PLoS One 9, e113287. doi: 10.1371/journal.pone.0113287 25405621 PMC4236181

[B64] Zhang Z. Ersoz E. Lai C.-Q. Todhunter R. J. Tiwari H. K. Gore M. A.. (2010). Mixed linear model approach adapted for genome-wide association studies. Nat. Genet. 42, 355–360. doi: 10.1038/ng.546 20208535 PMC2931336

[B65] Zhu C. Gore M. Buckler E. S. Yu J. (2008). Status and prospects of association mapping in plants. Plant Genome J. 1, 5–205. doi: 10.3835/plantgenome2008.02.0089

